# Alteration of Flt3-Ligand-dependent *de novo* generation of conventional dendritic cells during influenza infection contributes to respiratory bacterial superinfection

**DOI:** 10.1371/journal.ppat.1007360

**Published:** 2018-10-29

**Authors:** Ranin Beshara, Valentin Sencio, Daphnée Soulard, Adeline Barthélémy, Josette Fontaine, Thibault Pinteau, Lucie Deruyter, Mohamad Bachar Ismail, Christophe Paget, Jean-Claude Sirard, François Trottein, Christelle Faveeuw

**Affiliations:** 1 Univ. Lille, U1019 - UMR 8204 - CIIL - Centre d'Infection et d'Immunité de Lille, Lille, France; 2 Centre National de la Recherche Scientifique, UMR 8204, Lille, France; 3 Institut National de la Santé et de la Recherche Médicale U1019, Lille, France; 4 Centre Hospitalier Universitaire de Lille, Lille, France; 5 Institut Pasteur de Lille, Lille, France; 6 Laboratoire Microbiologie Santé et Environnement (LMSE), Ecole Doctorale des Sciences et de Technologie, Faculté de Santé Publique, Université Libanaise, Tripoli, Lebanon; University of Rochester Medical Center, UNITED STATES

## Abstract

Secondary bacterial infections contribute to the excess morbidity and mortality of influenza A virus (IAV) infection. Disruption of lung integrity and impaired antibacterial immunity during IAV infection participate in colonization and dissemination of the bacteria out of the lungs. One key feature of IAV infection is the profound alteration of lung myeloid cells, characterized by the recruitment of deleterious inflammatory monocytes. We herein report that IAV infection causes a transient decrease of lung conventional dendritic cells (cDCs) (both cDC1 and cDC2) peaking at day 7 post-infection. While triggering emergency monopoiesis, IAV transiently altered the differentiation of cDCs in the bone marrow, the cDC1-biaised pre-DCs being particularly affected. The impaired cDC differentiation during IAV infection was independent of type I interferons (IFNs), IFN-γ, TNFα and IL-6 and was not due to an intrinsic dysfunction of cDC precursors. The alteration of cDC differentiation was associated with a drop of local and systemic production of Fms-like tyrosine kinase 3 ligand (Flt3-L), a critical cDC differentiation factor. Overexpression of Flt3-L during IAV infection boosted the cDC progenitors’ production in the BM, replenished cDCs in the lungs, decreased inflammatory monocytes’ infiltration and lowered lung damages. This was associated with partial protection against secondary pneumococcal infection, as reflected by reduced bacterial dissemination and prolonged survival. These findings highlight the impact of distal viral infection on cDC genesis in the BM and suggest that Flt3-L may have potential applications in the control of secondary infections.

## Author summary

Secondary bacterial infections constitute a leading cause of mortality during influenza epidemics and pandemics. Acute infections trigger the mobilization of myeloid cells from the bone marrow (BM) to the infected tissues, a process referred to as emergency myelopoiesis. Influenza infection leads to a profound alteration in the pulmonary myeloid cell compartment characterized by the recruitment of inflammatory myeloid cells. The consequences of IAV infection on BM myelopoiesis have yet to be described in detail. Here, we report that influenza infection leads to reduced number of conventional dendritic cells (cDC) in the lungs (with a peak at day 7) and severely impairs DC differentiation in the BM, for the benefit of monopoiesis. The altered cDC differentiation was independent of pro-inflammatory cytokines and was not due to an intrinsic dysfunction of cDC precursors. Defective DC differentiation during influenza was associated with a decrease in the production of the key cDC differentiation factor, Fms-like tyrosine kinase 3 ligand (Flt3-L). Overexpression of Flt3-L during influenza enhanced the number of BM cDC progenitors and restored lung cDC compartment. This phenomenon was associated with a lowered number of lung inflammatory monocytes and reduced lung damages. Importantly, Flt3-L overexpression partially protected against secondary pneumococcal infection characterized by reduced bacterial loads, improved pathological outcomes and prolonged survival. Our results provide new clues as to how influenza infection influences the differentiation of cDCs, and highlight the potential therapeutic value of Flt3-L in a disease in desperate need of better treatments.

## Introduction

Influenza A viruses (IAV) are responsible for acute respiratory tract diseases and represent a threat to human health worldwide. Infection with IAV is frequently complicated by secondary bacterial infections; these typically occur between 4 to 14 days after the primary IAV infection (depending on the pathogenicity of the virus and the host’s immune system). In the context of influenza, physical disorders (e.g. impaired barrier function) and immunological disorders are the two main causes of enhanced susceptibility to bacterial infections (for reviews, [1, 2]). Upon IAV infection, the proportion and number of myeloid cells in the lungs change markedly. Indeed, inflammatory monocytes and monocyte-derived dendritic cells (DCs) are strongly recruited. Although monocyte-derived DCs are important for locally boosting the IAV-specific CD8^+^ T cell response [3–6], inflammatory monocytes exert a harmful pro-inflammatory effect in the lungs [7–10]. Regarding pulmonary sentinel cells, whether alveolar macrophages are depleted or not upon IAV infection remains an open question [6, 11, 12]. A recent study suggested that alveolar macrophages depletion/persistence upon IAV infection is largely dependent of mouse genetic background and infection conditions [13]. The situation is less clear for conventional DCs (cDCs), a family of critical sentinel cells composed by two main subpopulations in the lungs, namely cDC1 and cDC2. We recently reported a drastic decrease of total cDCs in IAV-infected mice [14]. Prior observations rather suggested a recruitment of cDC2 upon infection [4, 5, 11]. Regarding cDC1, whether they are depleted or not during the course of IAV infection remains unclear [4, 5, 11]. Differences in immunophenotyping strategies and in experimental design (*i*.*e*., viral dose/strain or mouse genetic background) could account for contrasting conclusions.

Myeloid lineage cells are derived from hematopoietic stem and progenitor cells in the bone marrow (BM). Studies in the mouse have provided a description of the successive steps leading to the generation of progenitors that give rise to both CD11b^+^ (cDC2) and CD8α^+^/CD103^+^ (cDC1) cDC subsets and monocytes/macrophages [15–17]. The macrophage/DC progenitors (MDPs) lose their ability to generate granulocytes but can develop into the common monocyte progenitors (cMoPs), restricted to monocytes and their descendants [18], or into common DC progenitors (CDPs). The CDPs further differentiate locally into plasmacytoid DCs and pre-DCs. Recent research has evidenced a high level of heterogeneity within the pre-DC population [19–21]. Importantly, these studies clearly showed that the BM contained pre-DC subsets having committed to cDC1 or cDC2 lineages. The pre-DCs subsequently leave the BM, enter the bloodstream, and migrate to peripheral tissues, where they give rise to cDC1 and cDC2 subsets [22–25].

The differentiation of DCs and monocytes in the BM is tightly controlled by growth factors, cytokines and transcription factors (for reviews [16, 26, 27]). Acute microbial infections induce a host phenomenon known as emergency myelopoiesis, which maintains the supply of myeloid cells (particularly monocytes) to the infected tissues. For example, systemic bacterial infection with *Ehrlichia muris*, *Yersinia Enterocolitica* or *Listeria monocytogenes* modulates myeloid progenitors in the BM, promotes myeloid cell differentiation, and thus contributes to host defences [28–31]. Systemic viral infections can also trigger myelopoiesis in the BM [32]. There is also evidence to suggest that local (i.e. non-systemic) infections can also indirectly affect BM myelopoiesis. For instance, intestinal infection with *Toxoplasma gondii* can act remotely to reprogram myeloid progenitors in the BM - leading to profound changes in monocyte functions [33]. Respiratory viral infections also trigger myeloid cell production in the BM, which influences lung immunity and contributes to viral clearance [34–38]. This emergency response to systemic or local infection is mediated by inflammatory mediators (*e*.*g*. type I and II interferons (IFN)) produced systemically [29, 30, 32, 34, 37, 39] or locally in the BM [33].

Very few studies have focused on the impact of IAV infection on emergency myelopoiesis in the BM. Early studies reported an increased number of mature (CD11b^+^Gr1^+^) myeloid cells in the BM of IAV-infected animals [34, 35, 38]. The increase in myelopoiesis during IAV infection seemed to be regulated by CD137-CD137L interaction in the BM [38]. However, Stiefter *et al*. recently reported that IFN signalling does not have a major role in regulating central myelopoiesis during infection [40]. In the current study, we report that IAV infection transiently affects the number of cDCs (both cDC1 and cDC2) in the lung compartment. We show for the first time that IAV reduced the number of BM progenitors committed to the DC lineage, i.e. CDPs, pre-DCs and, most importantly, the cDC1-biased pre-DC lineage. This reduction was associated with an increase in the production of Ly6C^hi^ monocytes in the BM. The phenomenon was independent of pro-inflammatory cytokines, and was associated with a reduction in the production of Fms-like tyrosine kinase 3 ligand (Flt3-L). Overexpression of Flt3-L during influenza infection enhanced the number of cDC progenitors in the BM, restored the cDC pool in the lungs, reduced lung damages and partially protected against secondary bacterial infections as reflected by decreased bacterial counts (reduced systemic dissemination) and prolonged survival.

## Results

### Influenza A virus infection triggers a drop of cDCs in the lungs and strongly alters the generation of cDC progenitors in the BM

**Fig 1:**
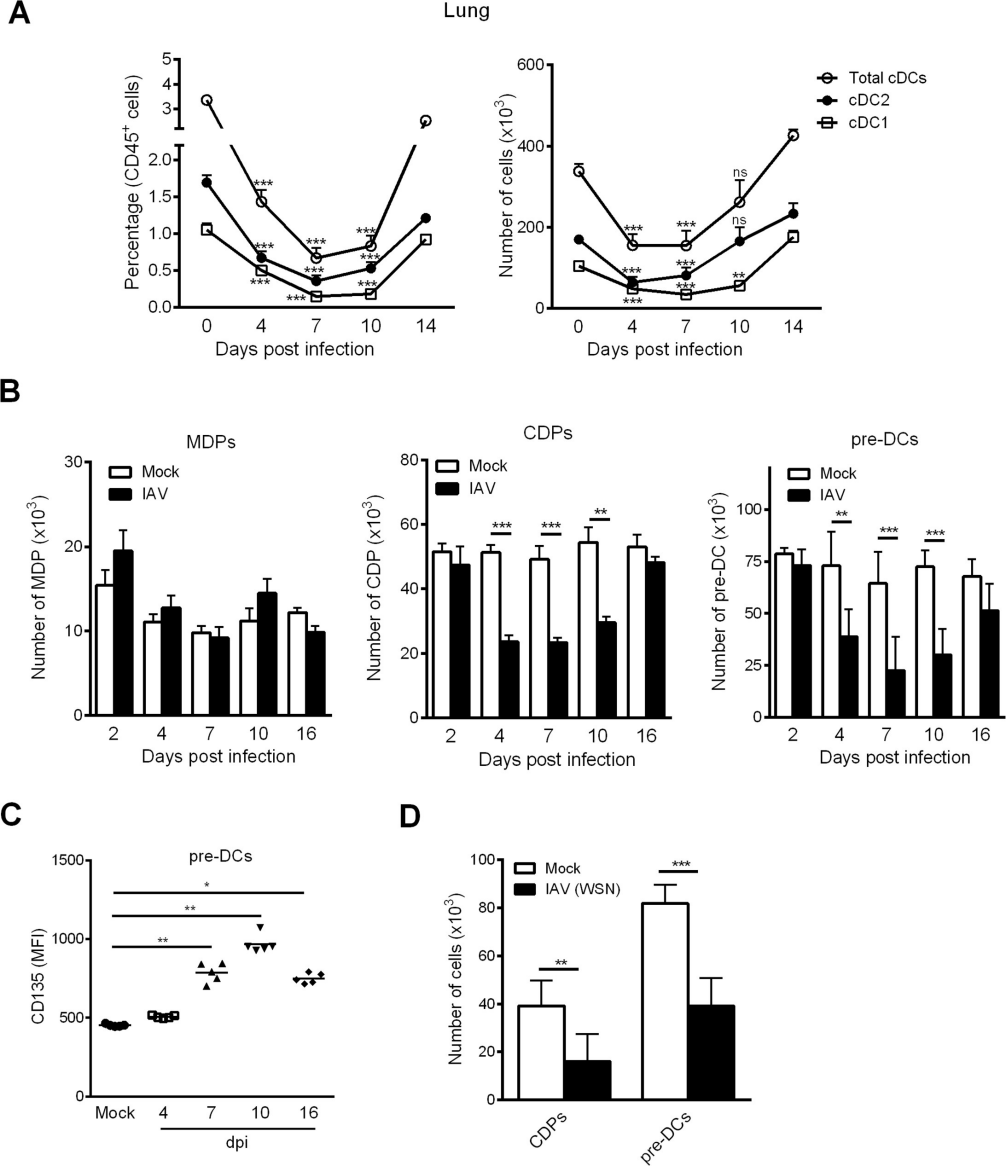
Influenza A virus infection leads to a time-dependent decrease of lung cDCs and DC progenitors in the BM. (A) Mice were infected i.n. with H3N2 virus. The frequency (*left panel*) and absolute number (*right panel*) of lung total cDCs (Siglec-F^-^CD11c^+^MHCII^+^CD64^-^), cDC2 (CD172α^+^CD24^low^) and cDC1 (CD172α^-^CD24^high^) subsets were assessed by flow cytometry at different time points post-infection. Means ± SEM from two experiments (n = 11) are represented. (B) Mock-treated and IAV-infected mice were sacrificed at different time point post-infection and the absolute number of BM MDPs, CDPs and pre-DCs were quantified by flow cytometry. Means ± SEM of biological replicates from at least three independent experiments are represented (n = 6–15). (C) The mean of fluorescent intensity (MFI) of CD135 expression on BM pre-DCs was analyzed at different time point post-infection. Data were pooled from two independent experiments (n = 6–8). (D) Mice were infected i.n. with WSN (H1N1) virus. The absolute number of CDPs and pre-DCs were assessed by flow cytometry at 7 dpi. Means ± SEM from two experiments (n = 10) were shown. *, p < 0.05; **, p < 0.01; ***, p < 0.001.

It has previously been reported that IAV infection affects the number of DCs in the lung compartment; this phenomenon might influence the disease outcome [4, 14]. However, whether cDCs - cDC1 and cDC2 - are recruited or reduced upon IAV infection remains an open question. We first sought to revisit prior findings using CD64, a marker that allows one to distinguish between cDCs and monocyte-derived DCs [41]. As shown in Fig 1A (the gating strategy is shown in S1A Fig), the frequency and absolute number of cDCs in the lungs of IAV (H3N2)-infected mice significantly decreased as early as 4 days post-infection (dpi) and peaked at 7 dpi. These changes were observed for both cDC1 (CD24^high^) and cDC2 (CD172α^+^) subsets. The number (but not the frequency) of cDCs and cDC2 returned to basal levels as early as 10 dpi whereas the number of cDC1 remained low at 10 dpi and returned to basal levels at 14 dpi (Fig 1A, *right panel*).

**Fig 2:**
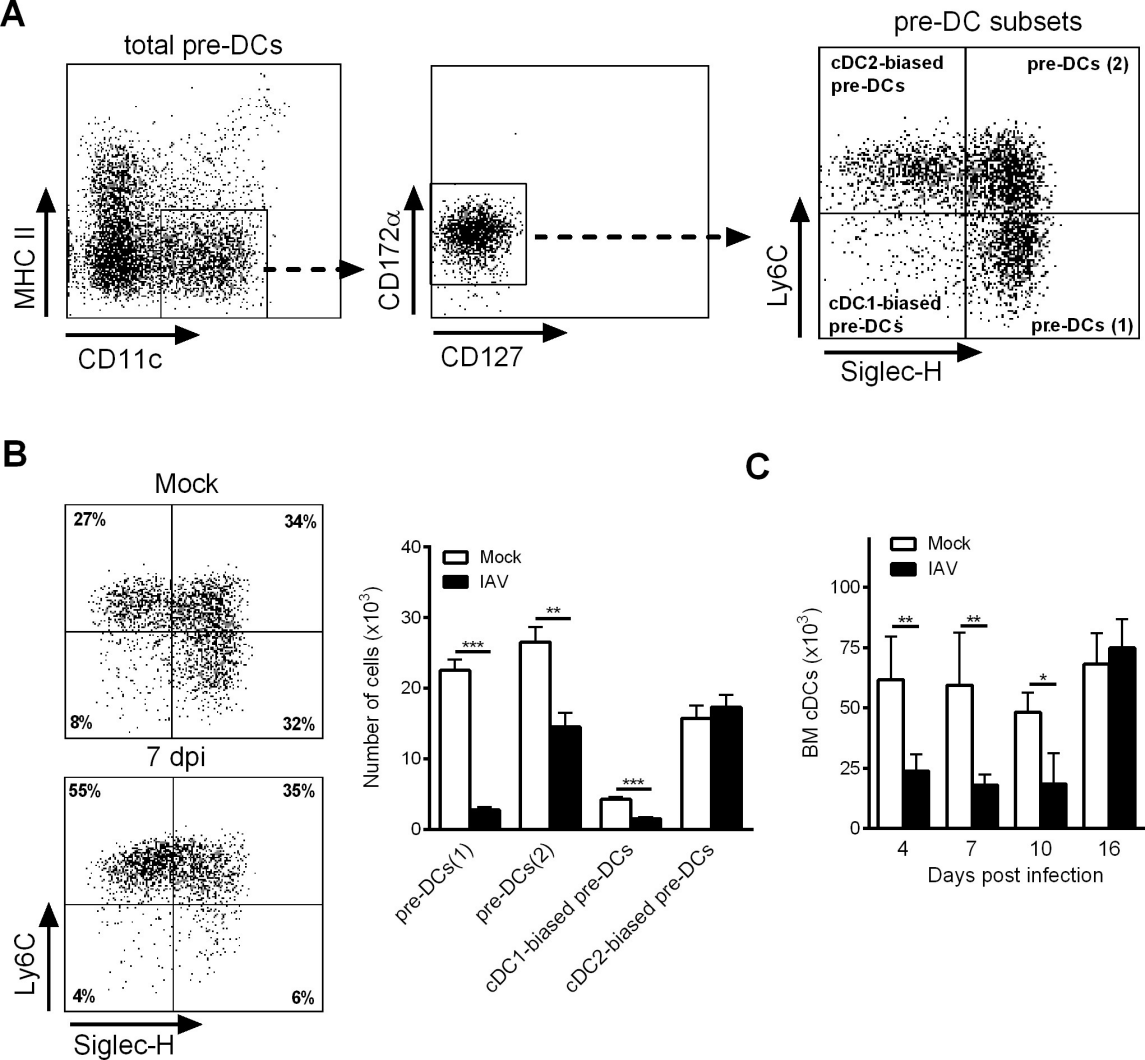
Influenza A virus infection affects pre-DC subset differentiation in the BM. (A) Gating strategy for BM pre-DC subset according to Siglec-F and Ly6C expression. (B) Mice were infected or not, with H3N2 virus and BM pre-DC subsets were analyzed at 7dpi. A representative dot plot was shown (*left panel*) and the absolute number of each pre-DC subset was then calculated (*right panel*). Means ± SEM of biological replicates from three experiments are represented (n = 12). (C) Mock-treated and IAV-infected mice were sacrificed at different time point post-infection and the absolute number of BM cDCs was quantified by flow cytometry (mean ± SEM, n = 5–8). *, p < 0.05; **, p < 0.01; ***, p < 0.001.

We next investigated influenza’s potential effect on the *de novo* generation of cDCs. To this end, we quantified DC progenitors in the BM over the course of IAV infection. As shown in Fig 1B (gating strategy shown in S1B Fig), the absolute number of MDPs did not change during infection, whereas the absolute CDP and pre-DC numbers were significantly lower between 4 dpi and 10 dpi. Interestingly, the remaining pre-DCs expressed higher levels of CD135 (the Flt3-L receptor) between 7 dpi and 16 dpi (Fig 1C). The CDP and pre-DC numbers returned to basal levels at 16 dpi. It is noteworthy that the numbers of CDPs and pre-DCs in the BM also fell during infection with H1N1 IAV (Fig 1D). This finding indicates that the altered differentiation of cDCs in the BM is a general consequence of IAV infection, regardless of the viral subtype. As reported recently, the BM’s pre-DC population is heterogeneous, and four subsets can be identified according to the cell surface expression of Siglec-H and Ly6C (Fig 2A) [21]. Siglec-H^+^Ly6C^-^ pre-DCs (1) differentiate into Siglec-H^+^Ly6C^+^ pre-DCs (2), which in turn give rise to cDC1- (Siglec-H^-^Ly6C^-^) or cDC2-biased pre-DCs (Siglec-H^-^Ly6C^+^). As shown in Fig 2B, the absolute numbers of pre-DCs (1), pre-DCs (2), and cDC1-biased pre-DCs decreased markedly at 7 dpi, whereas the number of cDC2-biased pre-DCs remained constant. Concomitantly with the changes in CDP and pre-DC counts, the number of cDCs in the BM also fell markedly between 4 dpi and 10 dpi (Fig 2C). Overall, IAV infection affects the number of cDCs in the lung tissue and significantly modifies the generation of DC precursors in the BM.

### Influenza A virus infection does not enhance DC precursor apoptosis or egress from the BM

**Fig 3:**
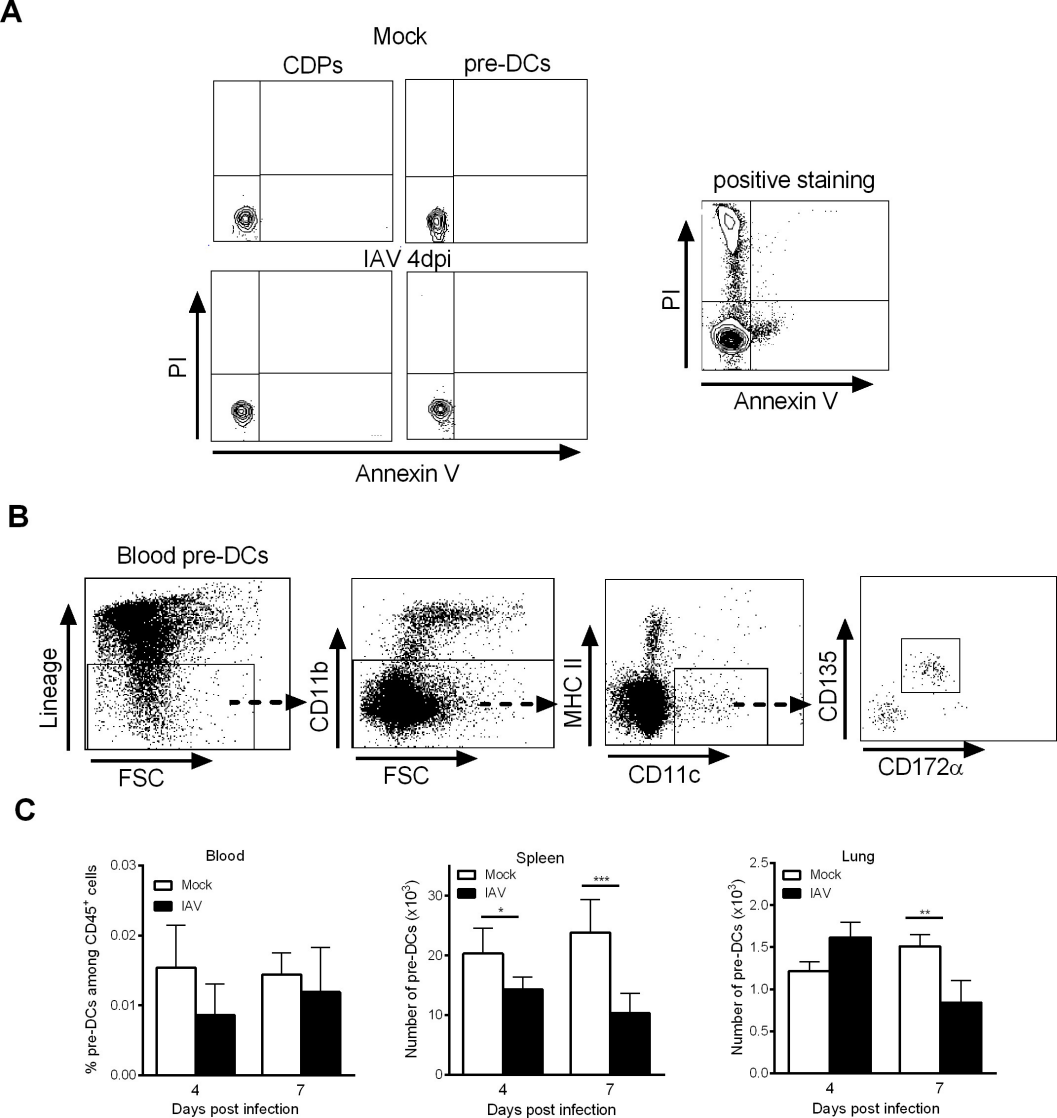
Influenza A virus infection does not lead to DC progenitor apoptosis or egress. (A) Representative dot plots of BM CDPs and pre-DCs from mock-treated and IAV-infected mice (4dpi) labelled with annexin V and iodide propidium are shown (*left panels*). Bone marrow cells left several hours at 37°C in PBS are positively stained for Annexin V and iodide propidium (positive staining, *right panel*). (B, C) Mice were infected i.n. with H3N2 virus and pre-DCs were quantified in peripheral tissues. (B) Example of gating strategy for blood pre-DC analysis. (C) The frequency of blood pre-DCs (*left*) and absolute number of spleen and lung pre-DCs (*middle and right*) were assessed by flow cytometry at 4dpi and 7dpi. Means ± SEM of 6–12 mice are represented. *, p < 0.05; **, p < 0.01.

We hypothesized that the change in the number of DC progenitors in the BM during IAV infection could be due to enhanced local apoptosis and/or egress. Whenever the time points analyzed (4 dpi is shown), the CDPs and pre-DCs were not stained by propidium iodide and annexin V (Fig 3A), suggesting that local apoptosis was not increased. Pre-DCs are known to migrate continually from the BM via the bloodstream to peripheral tissues like the spleen, where they serve as a reservoir for cDC development/turnover [24]. We therefore analyzed the pre-DCs in the blood, spleen and lungs of mock-treated and IAV-infected animals (Fig 3B and S2 Fig). As shown in Fig 3C, the frequency of pre-DCs in the blood did not fall significantly after the onset of IAV infection. Moreover, an analysis of pre-DCs in peripheral tissues revealed a significant decrease in the number of pre-DCs in the spleen (at 4 dpi) and in the lungs (at 7 dpi). It is noteworthy that during IAV infection, CDPs cannot be detected in the bloodstream or the spleen. These results suggest that the low number of cDC progenitors in the BM seen during IAV infection is not associated with an enhanced egress towards peripheral tissues.

### Influenza A virus infection does not lead to intrinsic dysfunction of DC precursors but affects the synthesis of DC differentiation factors

**Fig 4:**
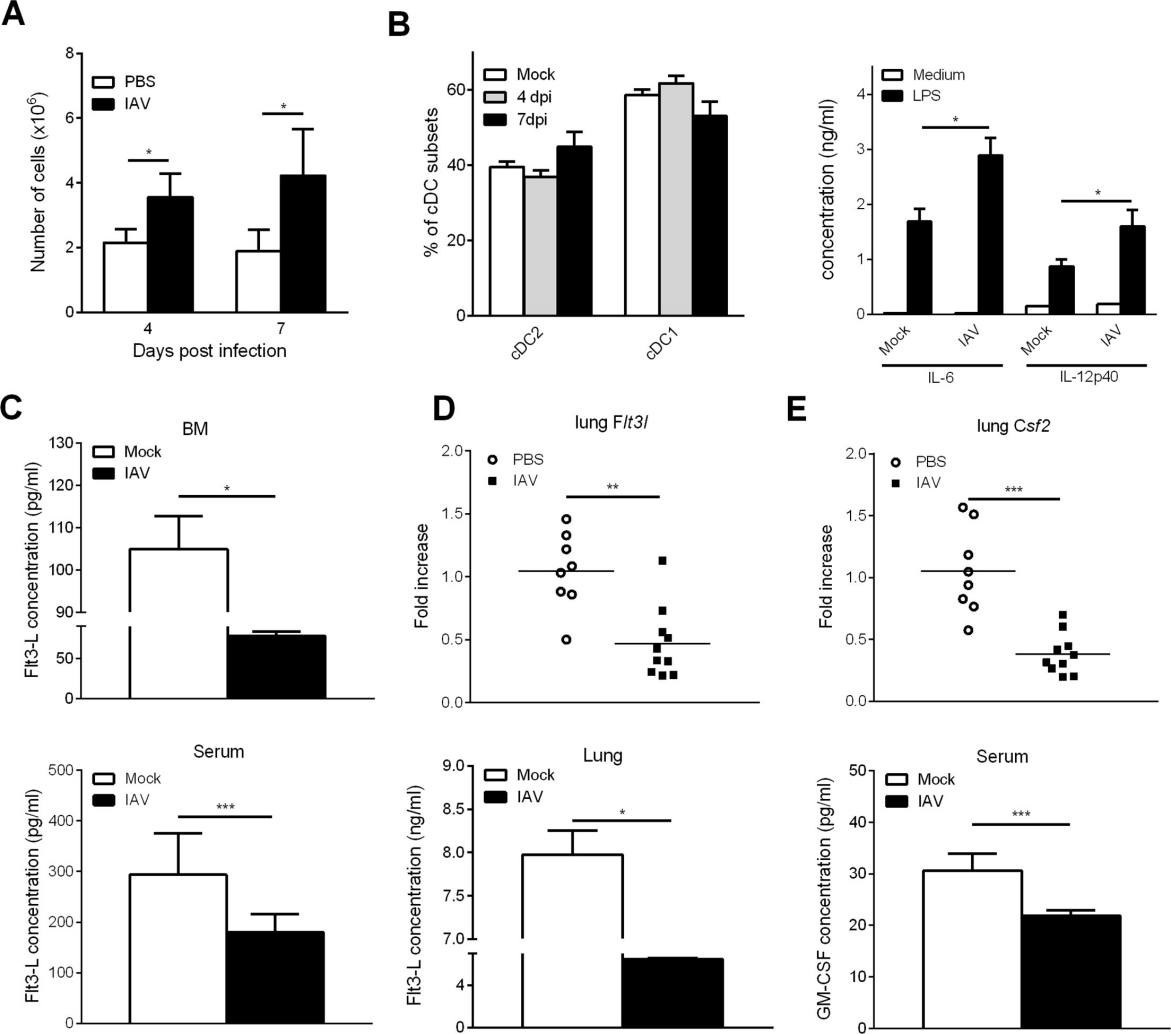
Influenza A virus infection does not lead to DC progenitor intrinsic dysfunctions. (A-B) Mice were infected (or not) i.n. with H3N2 virus and sacrificed at 4dpi and 7dpi. Bone marrow cells were cultured for 9 days with recombinant Flt3-L (100ng/ml). **(A)** Bone marrow-derived cells were enumerated and (B, *left panel*) the frequency of cDC subsets was assessed by flow cytometry. (B, *right panel*) BM-derived cells were stimulated with LPS (100ng/ml) for 24h and cytokine production was quantified in the supernatant. Means ± SEM of 4 mice are represented. (C) Production of Flt3-L was quantified in the BM protein extracts (*upper panel*) and in the serum (*lower panel*). Means ± SEM of biological replicates from four experiments are represented (n = 17). (D) mRNA copy numbers of *Flt3l (upper panel)* and Flt3-L production *(lower panel)* in the lung were assessed by quantitative RT-PCR or ELISA, respectively. (E) mRNA copy numbers of *Csf2* were determined by quantitative RT-PCR (*upper panel*) and GM-CSF production in the serum was quantified by ELISA (*lower panel*). (D, E) For quantitative RT-PCR, data were normalized to expression of *Gapdh* and are expressed as relative expression. Results shown are the individuals and means of 8–10 mice/group *, p < 0.05; **, p < 0.01; ***, p < 0.001.

To investigate whether influenza infection intrinsically affects the differentiation capacity of DC progenitors, BM cells from mock-treated and IAV-infected (4 dpi and 7 dpi) mice were cultured *in vitro* in the presence of Flt3-L, a key factor necessary for cDC differentiation. Relative to mock-treated mice, an increased number of Flt3-L-derived cells was generated from the BM of IAV-infected mice (Fig 4A). Flow cytometry analysis revealed that around 10% of these cells were plasmacytoid DCs and 80% were cDCs (S3A Fig). Of note, on the remaining cells, we did not detect any staining with anti-CD115 and anti-CD11b monoclonal antibodies (Abs) suggesting the absence of monocytes (S3A Fig). The frequencies of cDC1 (CD172α^+^CD24^high^) and cDC2 (CD172α^-^CD24^low^) subsets (gating strategy in S3A Fig) were unchanged between mock-treated and IAV-infected mice (Fig 4B, *left panel*). Finally, upon LPS stimulation, DCs generated from IAV-experienced BM cells produced higher amounts of IL-6 and IL-12p40 relative to controls (Fig 4B, *right panel*); the IL-10 production being equivalent in both groups (S3B Fig). Thus, DC progenitors from IAV-infected animals do not display major intrinsic disorders that could block their differentiation.

We next hypothesized that influenza could interfere with the production of differentiation factors crucial for the differentiation of cDC. As shown in Fig 4C, the concentration of Flt3-L in the BM (*upper panel*) and in the blood (*lower panel*) was significantly decreased at 7 dpi. Because the Flt3-L/Flt3 pathway is also critical for pre-DC differentiation towards mature cDCs in peripheral tissues, including the pulmonary compartment [42], Flt3-L production was quantified in the lungs. Remarkably, a decreased expression of *Flt3l* transcript and Flt3-L protein was noticed in the lung of infected animals (7 dpi) (Fig 4D). Together with Flt3-L, granulocyte/macrophage colony-stimulating factor (GM-CSF) is also critical for non-lymphoid tissue cDC homeostasis [43]. As shown in Fig 4E, relative to mock-treated animals, the expression of *Csf2* transcripts in the lungs and GM-CSF levels in the serum were significantly decreased in IAV-infected animals. Hence, IAV infection alters the production of key factors for cDC differentiation, namely Flt3-L and GM-CSF.

### Influenza A virus infection leads to increased monopoiesis in the BM

**Fig 5:**
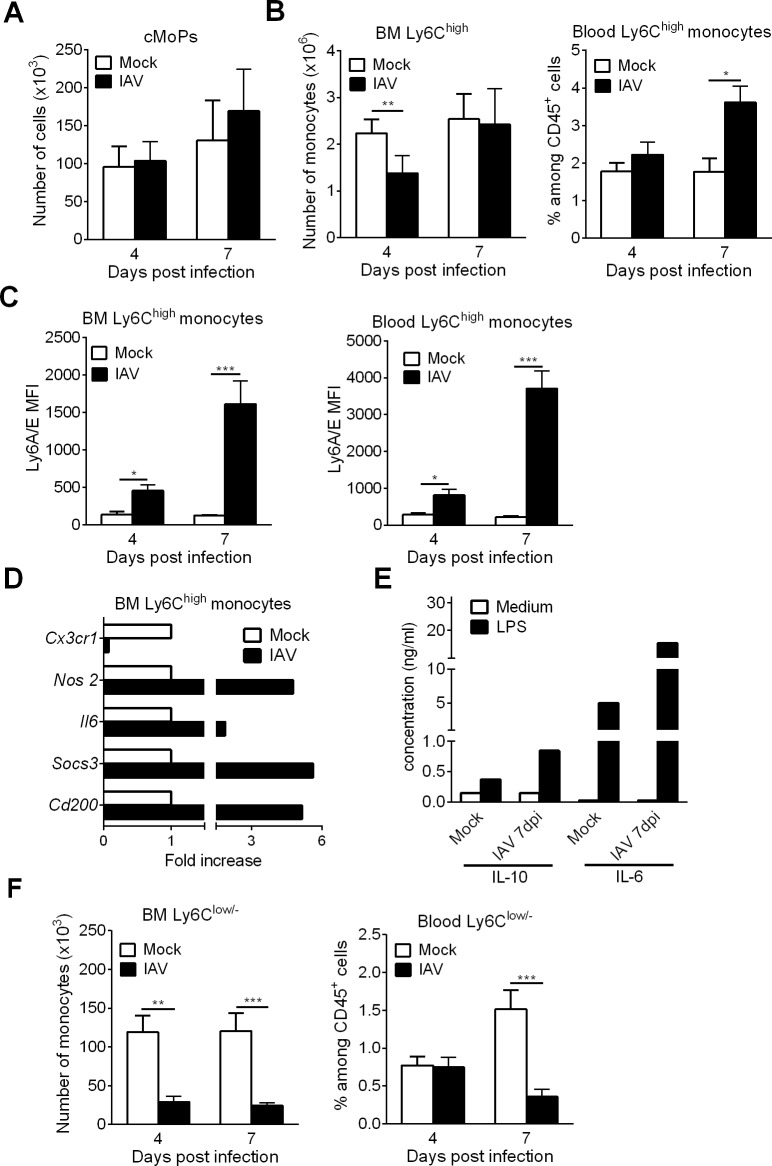
Influenza A virus infection increases monopoiesis in the BM. (A-C) Mock-treated and IAV-infected mice were sacrificed at 4dpi and 7dpi. (A) The absolute number of BM cMoPs was quantified by flow cytometry. Means ± SEM of biological replicates from two experiments are represented (n = 6). (B) The absolute number of BM CD115^+^Ly6C^+^ monocytes *(left panel*) and the frequency of blood BM CD115^+^Ly6C^+^ monocytes (among CD45^+^ cells) *(right panel*) were assessed by flow cytometry. Data were pooled from three experiments (n = 9). (C) The mean of fluorescent intensity (MFI) of Ly6A/E expression on BM and blood Ly6C^+^ monocytes was analyzed. Data were pooled from two experiments (n = 6–7). (D-E) Ly6C^high^ monocytes were FACS sorted from BM (pool of 3 mice) of mock-treated and IAV-infected animals (7dpi). (D) mRNA copy numbers of pro- and anti-inflammatory genes were determined by quantitative RT-PCR. Data are normalized to expression of *Gapdh* and are expressed as fold increase over average gene expression in mock animals. Data represent the means ± SEM of biological replicates (n = 2) (E) Purified monocytes were cultured for 24h with LPS (100ng/ml) and cytokine production was quantified by ELISA. Means of biological duplicates are shown. (F) The absolute number of BM CD115^+^Ly6C^low/-^ monocytes *(left panel*) and the frequency of blood BM CD115^+^Ly6C^low/-^ monocytes *(right panel*) were assessed by flow cytometry. Means ± SEM of biological replicates from three experiments are represented (n = 9). *, p < 0.05; **, p < 0.01; ***, p < 0.001.

A recent study found that in the context of a systemic bacterial infection, the immune system responds by promoting emergency monopoiesis at the expense of DC differentiation [30]. We therefore looked at the influence of IAV infection on monopoiesis. In IAV-infected mice, the numbers of cMoPs (the direct progeny of MDPs) in the BM did not change significantly at any time point (Fig 5A). In stark contrast, the number of Ly6C^high^ monocytes (often referred to as “inflammatory monocytes”) in the BM fell significantly at 4 dpi but not at 7 dpi (Fig 5B, *left panel*). These changes were associated with an elevated number of Ly6C^high^ monocytes in the blood at 7 dpi, which is suggestive of enhanced egress from the BM (Fig 5B, *right panel*). The expression of Ly6A/E (also known as Sca-1), a marker normally found on hematopoietic stem cells, on inflammatory monocytes in the BM and the blood was significantly higher in IAV-infected mice than in mock-treated mice (Fig 5C). To gain further insight into the immunological features of Ly6C^high^ monocytes, cells were sorted from the BM of control and IAV-infected mice (7 dpi). Analysis of gene expression profiles revealed the upregulation of both pro-inflammatory genes (*Nos2* and *Il6*) and anti-inflammatory genes (*Socs3* and *Cd200*) and the drastic downregulation of *Cx3cr1* transcription (Fig 5D). This finding indicates that IAV triggers the generation of Ly6C^high^ monocytes with a regulatory phenotype, as recently reported by Askenase *et al*. [33]. Accordingly, Ly6C^high^ monocytes from IAV-infected mice produced more IL-6 and IL-10 than control mice following stimulation with LPS (Fig 5E). Lastly, IAV infection led to a significant fall in the number of Ly6C^low/-^ (patrolling) monocytes in the BM (at 4 dpi and 7 dpi) and the blood (at 7 dpi) (Fig 5F). Taken as a whole, these data suggest that IAV infection triggers emergency monopoiesis at the expense of DC differentiation.

### Altered DC differentiation does not depend on pro-inflammatory cytokines

**Fig 6:**
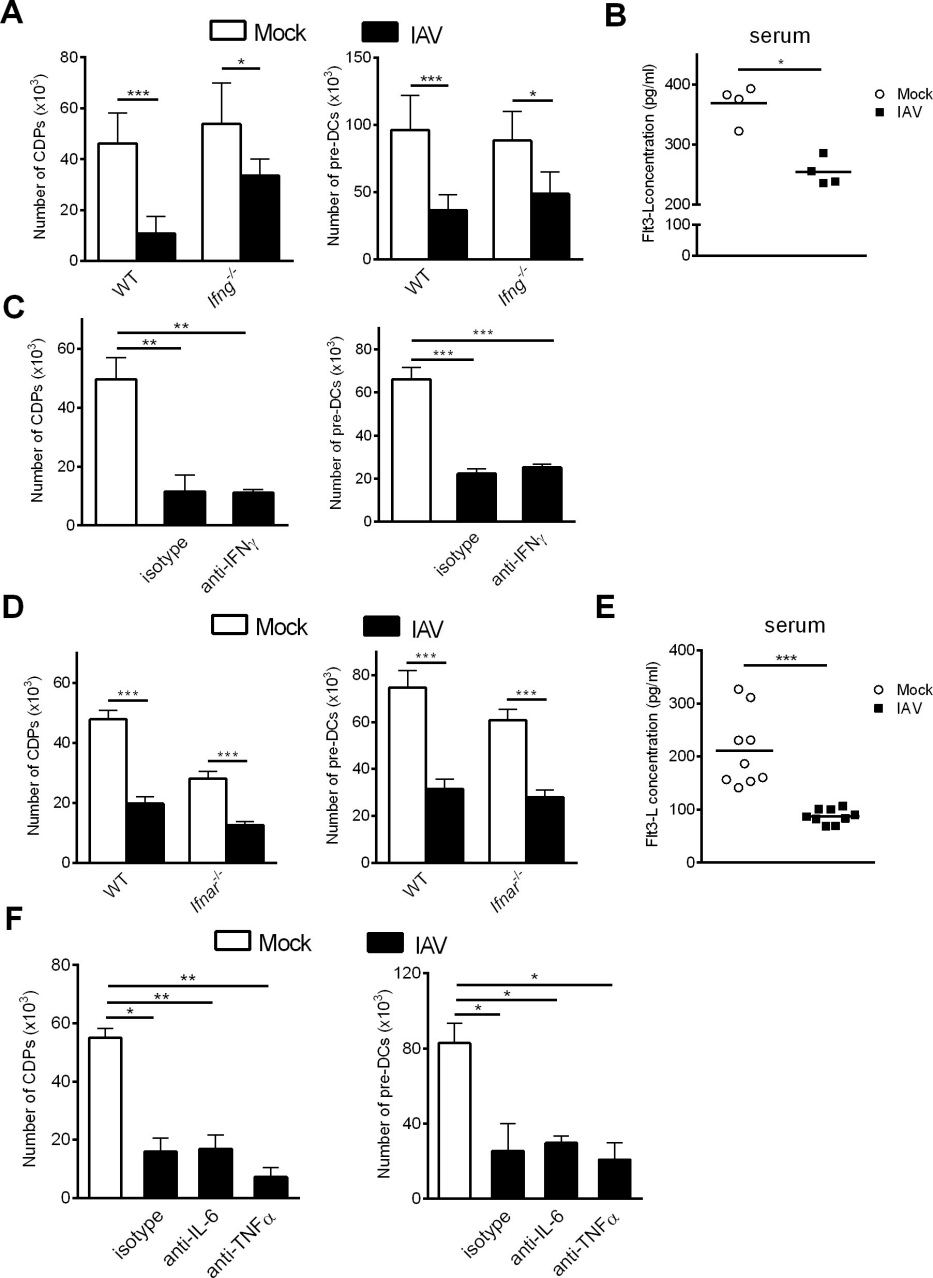
Neutralization of pro-inflammatory cytokines does not restore the DC progenitor compartment during IAV infection. (A) Age- and sex-matched WT and *Ifng*^-/-^ mice were infected (or not) i.n. with H3N2 virus. (B) Production of Flt3-L was quantified in the serum of *Ifng*^-/-^ mice infected or not with IAV (7 dpi) (n = 4). (C) Infected WT mice were treated at day 0, 2, 4 and 6 pi with anti-IFN-γ mAb or isotype control (300μg/mouse). (D) Age- and sex-matched WT and *Ifnar*^-/-^ mice were infected or not with IAV. (E) Production of Flt3-L was quantified in the serum of *Ifnar*^-/-^ mice infected or not with IAV (7 dpi) (n = 9). (F) Infected WT mice were treated at day 0, 2, 4 and 6 with anti-IL-6, anti-TNFα or isotype (300μg/mouse). (A, C, D, F) The absolute number of BM CDPs and pre-DCs were assessed by flow cytometry at 7dpi. (A, C) Means ± SEM of biological replicates from two or three experiments are shown (n = 9–12). (D) Data are pooled from three experiments (n = 10–15). (F) Means ± SEM of biological replicates are shown (n = 5). *, p < 0.05; **, p < 0.01; ***, p < 0.001.

We next sought to identify the molecular mechanisms underlying the fall in DC progenitors in the BM during influenza infection. It was recently reported that IFN-γ signalling impairs cDC development after systemic infection with *Y*. *Enterocolitica* [30]. To study the potential role of IFN-γ in our setting, *Ifng*^-/-^ mice were infected with IAV. As was the case in wild type (WT) mice, *Ifng*^-/-^ mice exhibited a significant decrease in CDP and pre-DC numbers at 7 dpi (Fig 6A). Of note, IFN-γ deficiency did not restore the drop of Flt3-L concentration in the sera (Fig 6B). Moreover, the treatment of IAV-infected mice with anti-IFN-γ monoclonal Ab did not restore the number of CDPs or pre-DCs, when compared with isotype-treated mice (Fig 6C). This suggests that the changes in cDC generation during IAV infection are IFN-γ independent.

Next, we assessed the role of other pro-inflammatory cytokines including type I IFNs, TNF-α, and IL-6, all of which are known to trigger emergency myelopoiesis, in altered DC differentiation [34, 37, 39, 44–46]. The abrogation of type I IFN signalling did not influence the numbers of CDPs and pre-DCs in the BM (Fig 6D). In the same line, the decreased serum level of Flt3-L observed in IAV-infected WT mice was also noticed in IAV-infected *Ifnar*^*-/-*^ animals (Fig 6E). Finally, neutralizing TNF-α and IL-6 activities did not restore the numbers of CDPs and pre-DCs during IAV infection (Fig 6F). Hence, the influenza-induced alteration in cDC differentiation is independent of IFN-γ, type I IFNs, TNF-α, and IL-6. Our data also suggest that Flt3-L production is not regulated by (type I and II) IFN signaling.

### Overexpression of Flt3-L during IAV infection boosts cDC differentiation in the BM, restores the cDC pool in the lungs and attenuates the recruitment of inflammatory monocytes

**Fig 7:**
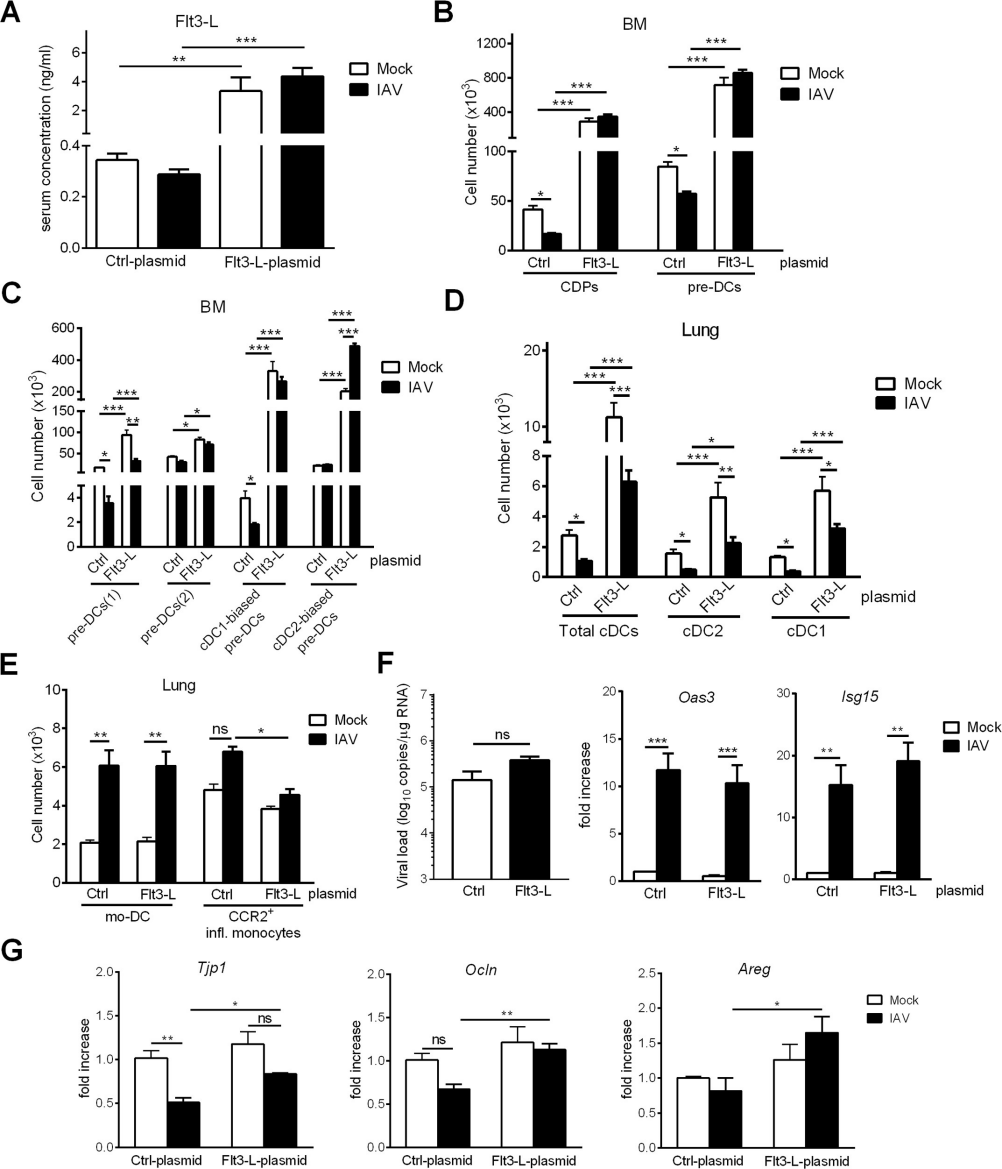
Flt3-L overexpression during IAV infection enhances cDC generation and is associated with an increased expression of genes involved in lung barrier integrity. Mice were i.v. injected with 2μg of a control- or Flt3-L- encoding plasmid. Twenty four hours later, mice were infected, or not, with IAV. Mice were sacrified at 4 dpi. (A) The production of Flt3-L was quantified in the sera, (B) the numbers of CDPs, total pre-DCs and (C) pre-DC subsets were assessed in the BM by flow cytometry. (D) The numbers of cDC subsets and (E) other myeloid cells were determined in the lungs. (F)(*left panel*) Viral load (in log_10_ viral M1 RNA copies/μg RNA) and (*middle and right panel*) mRNA expression of *Oas3* and *Isg15* genes were quantified in the lungs (RT-PCR). (G) mRNA copy numbers of genes associated with the barrier integrity (*Ocln*, *Tjp1*) and tissue repair (*Areg*) were determined in the lungs (RT-PCR). All RT-PCR data are normalized to expression of *Gapdh* and are expressed as fold increase over average gene expression in Ctrl-plasmid/mock animals. Data represent the means ± SEM of biological replicates (A-E) n = 6–12 and (F-G) n = 5. *, p < 0.05; **, p < 0.01; ***, p < 0.001, ns: not significative.

Several studies have found that the immunosuppression arising after burns or sepsis can be corrected by restoring the DC compartment, e.g. by DC transfer or inoculation of Flt3-L [47–50]. We first looked at whether the cDC population could be restored during influenza infection. To this end, an Flt3-L-encoding plasmid was i.v. injected into animals one day before IAV infection in order to enhance systemic production of Flt3-L. As shown in Fig 7A, the serum Flt3-L concentration was higher in treated, mock and IAV-infected, mice than in control animals. An analysis of DC progenitors in the BM revealed an increased number of CDPs and total pre-DCs in Flt3-L-treated animals (Fig 7B). It is noteworthy that their numbers were similar in mock-treated and IAV-infected animals. Interestingly, Flt3-L overexpression was associated with a decreased CD135 expression on pre-DCs in both animal groups (S4 Fig). A pre-DC analysis highlighted elevated numbers of all subsets in response to Flt3-L regardless of infection, although the increase was greatest for the cDC1-biased preDCs (Fig 7C). Enhanced Flt3-L synthesis during influenza infection was also associated with an increased number of total cDCs (cDC1 and cDC2 subsets) in the lungs of mock-treated and IAV-infected animals (Fig 7D). It is noteworthy that, in contrast to the BM compartment, Flt3-L overexpression induced a greater expansion of cDC in mock-treated animals relative to IAV-infected animals. Administration of the Flt3-L-encoding plasmid did not influence the number of monocyte-derived DCs and neutrophils in the lung compartment (Fig 7E and S4 Fig). In contrast, and interestingly enough, Flt3-L overexpression significantly reduced the number of CCR2^+^ inflammatory monocytes upon IAV infection (Fig 7E). We next investigated whether restoration of the cDC compartment in the lungs affects viral replication. Overexpression of Flt3-L had a moderate and not significant effect on viral load (slightly enhanced) and did not impact the expression of IFN-stimulating genes (*i*.*e*. *Isg15*, *Oas3*), the expression of which being generally related to the viral burden (Fig 7F). A recent study from Ellis and colleagues showed that the early recruitment of CCR2^+^ inflammatory monocytes during IAV infection is critical in epithelial barrier damage and secondary bacterial invasion [7]. In line with the diminished number of CCR2^+^ inflammatory monocytes (Fig 7E), Flt3-L plasmid prevented the reduced gene expression of *Tjp1* (tight junction protein 1) and *Ocln* (occludin); these proteins being associated with lung barrier functions. In parallel, Flt3-L overexpression increased the transcript expression of *Areg (*amphiregulin), a protein that is essential for restoring epithelial integrity and tissue repair during influenza infection [51, 52]. Overall, Flt3-L overexpression during IAV infection boosted the BM cDC differentiation, restored the number of pulmonary cDCs and lowered the infiltration of inflammatory monocytes; the latter effect might explain higher expression of genes involved in lung barrier integrity.

### Flt3-L overexpression during IAV infection partially protects against secondary pneumococcal infection

**Fig 8:**
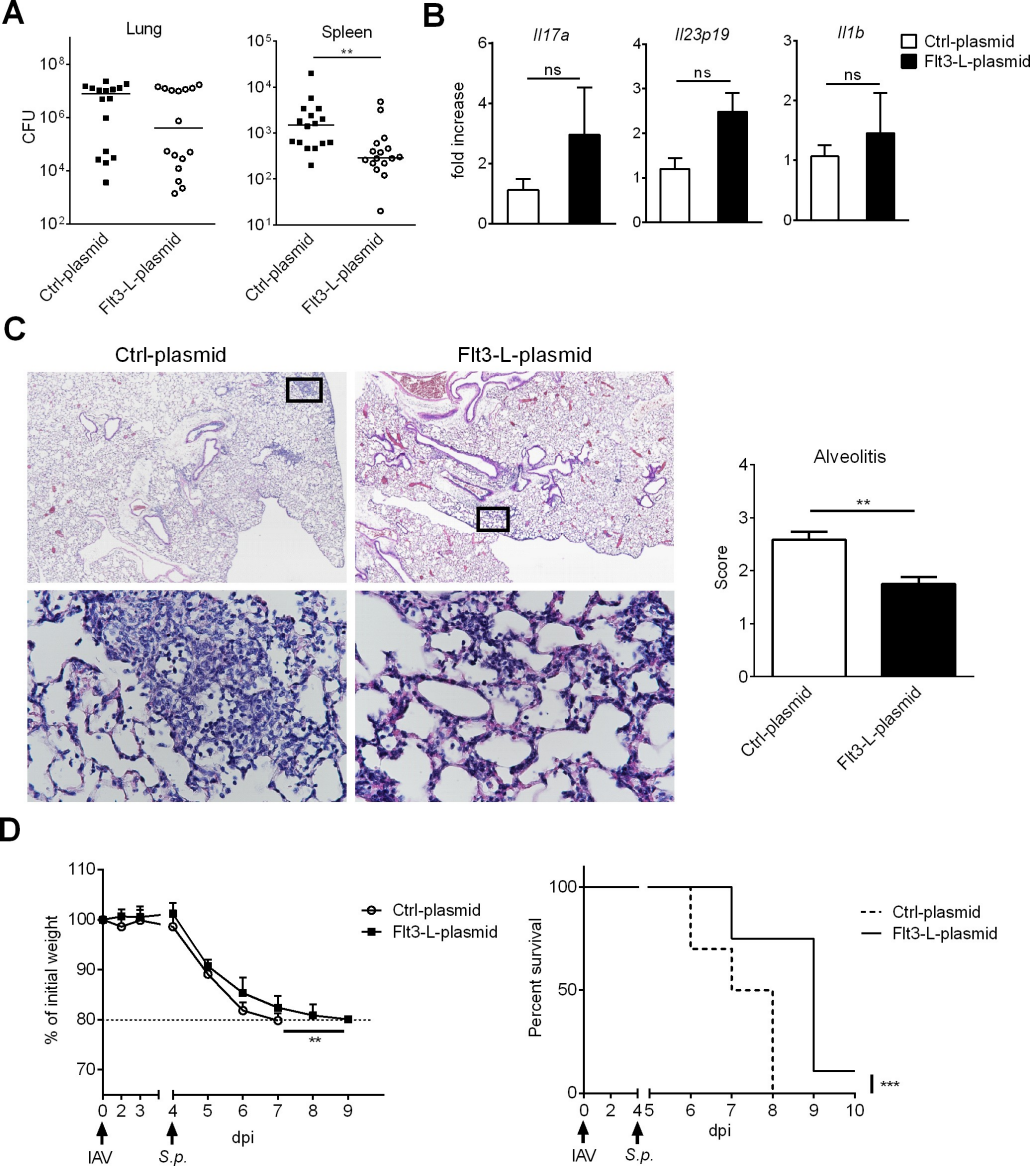
Flt3-L treatment during IAV infection partially protects against secondary pneumococcal infection. Mice were i.v. injected with 2μg of a control- or Flt3-L-encoding plasmid and 24h later, mice were infected with IAV. Four days post-influenza infection, mice were infected with *S*. *pneumoniae* (10^3^ CFUs). (A) Mice were sacrificed 30h after *S*. *pneumoniae* challenge and the number of CFUs was determined in the lungs and spleen. The solid lines correspond to the median values. Data represent pooled results from two independent experiments (n = 16). (B-C) Mice were sacrificed 12h after *S*. *pneumoniae* challenge. (B) mRNA copy numbers of genes encoding Th17-related cytokines were quantified by RT-PCR. All RT-PCR data are normalized to expression of *Gapdh* and are expressed as fold increase over average gene expression in Ctrl-plasmid/co-infected animals (n = 5). (C) Histological analysis of lung sections. *Left panel*: Representative lung sections stained with hematoxylin and eosin are shown. Diffuse alveolar inflammation is marked with interstitial thickening in Ctrl-plasmid-treated animals but remains moderate in Flt3-L-treated animals. Lung images are shown at x4 magnification (*upper panel*) and black squares in lung images are shown at x40 magnification (*lower panel*). *Right panel*: Blinded sections were scored for levels of alveolitis. Data represent the means ± SEM (n = 4 mice/group). (D) Body weight evolution (in % initial body weight) (means ± SD) and survival rate of co-infected mice treated or not with Flt3-L were monitored daily (n = 10). **, p < 0.01; ***, p < 0.001, ns: not significative.

We next sought to investigate whether Flt3-L overexpression during IAV infection could ameliorate the outcomes of secondary bacterial infections. To this end, Flt3-L-treated, IAV-infected mice were secondarily infected with *Streptococcus pneumoniae*, a major opportunistic pathogen implicated in bacterial superinfection. In this system, a low dose of *S*. *pneumoniae* (10^3^ colony forming units, CFUs) is sufficient for local bacterial outgrowth and systemic translocation; both bacterial pneumonia and bacteraemia contributing to death [1, 2]. Interestingly, albeit it did not reach significance, Flt3-L-treated mice had lower bacterial counts in the lungs than mice treated with the control plasmid (Fig 8A, *left panel*). Furthermore, Flt3-L overexpression led to a significant reduction in bacterial dissemination from the lungs, as revealed by the lower number of viable bacteria in the spleen (Fig 8A, *right panel*). Expression of Th17-related cytokines is important to control the outgrowth of respiratory bacteria in the context of prior influenza infection [53–55]. No significant differences in the expression of genes encoding for Th17-inducing cytokines (*Il19p23*, *Il1b*) and the Th17-related cytokine *Il17a* were observed in co-infected mice, either treated or not with Flt3-L (Fig 8B). To determine the effect of Flt3-L overexpression on pulmonary damage, we scored lung histology slides. All co-infected mice showed histological evidence of lung inflammation evidenced by interstitial inflammation and alveolar lesions (Fig 8C). Compared to the control group, Flt3-L-treated mice had less marked alveolar lesions, less diffuse alveolar inflammation (mainly composed of neutrophils and lymphocytes) and less interstitial thickening (scored in Fig 8C, *right panel*). Few bronchial lesions were observed at this time point and no major differences were noticed between the two groups. We then determined whether the positive effect of Flt3-L on bacterial loads and lung pathology extended to ameliorated morbidity and mortality outcomes. Indeed, after the bacterial challenge, co-infected mice that received the Flt3-L plasmid lost weight but less rapidly relative to the control group (Fig 8D, *left panel*). Interestingly, restoration of Flt3-L expression during IAV infection led to a significant delayed mortality compared to controls (Fig 8D, *right panel*). Taken as a whole, these results suggest that Flt3-L treatment during influenza infection partially protects against secondary bacterial infection as reflected by decreased bacterial counts (reduced systemic dissemination), reduced pneumonia and prolonged survival.

## Discussion

The regulation of DC homeostasis during infection is of major importance because DCs are essential for the initiation of innate and acquired immune responses. Many studies reported DC depletion during systemic infections such as sepsis (for a review, [56]). Along with impaired DC function, this DC depletion is a major cause of immunosuppression and predisposition to bacterial infections [57, 58]. In the present study, we investigated the impact of local (pulmonary) viral infection on cDC homeostasis. Our observation of a transient depletion of cDCs - cDC1 and cDC2 - in the lungs of IAV-infected mice contrasts with previous reports showing a recruitment of cDC2 and the absence or a slight decrease of cDC1 [4, 5, 11]. The methodology used in the current report, *i*.*e*. use of anti-CD64 mAb which discriminates monocyte-derived DCs and cDCs, differed from these prior studies and could explain the different conclusions. Importantly, we showed for the first time that IAV infection strongly affects cDC progenitor differentiation in the BM, a phenomenon associated with an impaired production of Flt3-L. Overexpression of Flt3-L during IAV infection enhanced cDC differentiation in the BM, restored the cDC counts in the lungs and lowered inflammatory monocytes’ infiltration and lung injury. Importantly, Flt3-L overexpression provided partial protection against secondary bacterial infection.

Very few studies have investigated the impact of IAV infection on BM cells *in vivo*. Sedger et *al*. reported a severe depletion of the B cell lineage during infection [59]. With regard to myelopoiesis, several studies have suggested that IAV infection promotes monopoiesis [34, 35, 38]. The large observed decrease in the number of cDCs in the lungs during IAV infection prompted us to look at whether this infection influences DC differentiation in the BM. We observed a severe, transient (4–10 dpi) decrease in the number of CDPs and pre-DCs but not MDPs. It is noteworthy that this period coincides with the peak of susceptibility to bacterial superinfection. We next characterized the molecular basis of this drastic decline in DC progenitors in the BM. In contrast to the B cell lineage [59], our results suggest that apoptosis of DC progenitors in the BM was not affected by IAV. Moreover, the observed reduction in pre-DC numbers in the blood, spleen and lungs argued against increased egress of DC progenitors from the BM, although further investigations regarding for instance the entry/retention of pre-DCs in peripheral tissues would be necessary to draw a firm conclusion. A similar decrease in the number of DC progenitors was recently described in a model of systemic *Y*. *enterocolitica* infection; the authors showed that infection favoured monopoiesis at the expense of DC differentiation in a Toll-like receptor 4 and IFN-γ-dependent manner [30]. Our results are in line with this report, and demonstrate that IAV favours monopoiesis at the expense of cDC development. In contrast to Pasquevich *et al*.’s report, however, the decrease in the CDP and pre-DC numbers during IAV infection did not depend on IFN-γ. This disparity might be due to the presence of *Y*. *enterocolitica* in the BM [30]. In contrast, IAV which is restricted to the respiratory tract, is barely detectable in the BM during infection [34], which suggests an indirect mode of action. We hypothesized that the pro-inflammatory cytokine storm following IAV infection is responsible for altering DC development in the BM. It has been reported that type I IFNs, IL-6 and TNF-α promote emergency monopoiesis in some settings [39, 60, 61]. Here, we found that the decrease in the number of CDPs and pre-DCs during IAV infection is independent of these cytokines. Although we cannot fully rule out the possibility of a combined mode of action, these pro-inflammatory cytokines do not appear to be important in the control of cDC generation during IAV infection. Type III IFNs represent an emerging family of cytokines that functions as a first-line of defence against influenza infection [62–64]. Although the impact of type III IFNs on myelopoiesis is presently unknown, it might be interesting to investigate its potential role (in combination or not with type I IFNs) during the course of IAV infection.

During IAV infection, the transient decrease in Ly6C^high^ monocytes in the BM and their concomitant increase in the blood is consistent with the known recruitment of inflammatory monocytes in the lungs of IAV-infected mice [7–10, 40]. Importantly, our analysis of the Ly6C^high^ monocytes’ phenotype and cytokine production highlighted the presence of a mixed regulatory/inflammatory population in the BM of infected animals. Monocyte function is in general shaped by local signals in the tissue where the cells are recruited. A recent study demonstrated that monocytes can also be pre-emptively educated during their development in the BM [33]. Additional studies will be required to establish whether monocyte progenitor cells can be programmed in the BM for regulatory functions during IAV infection. In parallel with the changes in Ly6C^high^ monocytes, we showed for the first time that an acute respiratory infection could lead to a drastic decrease in the numbers of BM and blood patrolling Ly6C^low/-^ monocytes. This finding is in line with the reported recruitment of Ly6C^low/-^ monocytes to the lungs of IAV-infected mice [40]. Lung Ly6C^low/-^ monocytes can be switched towards an inflammatory phenotype, a process controlled by type I IFNs [40]. The changes in Ly6C^low/-^ monocyte homeostasis in the BM during IAV infection could also be due to increased generation of inflammatory Ly6C^high^ monocytes at the expense of patrolling Ly6C^low/-^ monocytes. Both hypotheses are not mutually exclusive.

Influenza infection does not cause intrinsic functional defects of BM progenitors/precursors upstream of CDPs, i.e. MDPs. *In vitro*, BM cells collected from IAV-infected animals mainly differentiated into cDCs, including both cDC1 and cDC2 subsets, in the presence of Flt3-L. Interestingly, the number of cDCs generated *in vitro* from IAV-experienced BM cells was greater than BM cells from mock-treated animals. This process is probably due to the increased expression of Flt3 (CD135) on DC progenitors. In the present study, we observed a decrease in the production of Flt3-L in the BM, blood and lungs during IAV infection, a phenomenon that could account for the decrease in CDPs and/or pre-DCs. This finding contrasts with other pathological conditions where decreased DC number is associated with enhanced synthesis of Flt3-L [65–68]. In these contexts, Flt3-L promotes DC expansion. The decrease in Flt3-L production during IAV infection is counter-balanced by the enhanced expression of Flt3 on DC progenitors. The differentiation of pre-DCs into cDC1 and cDC2 subsets at peripheral sites requires Flt3-L [22]. During IAV infection, it is likely that low Flt3-L production in the lung affects the *in situ* differentiation of pre-DCs towards cDC1 and cDC2 subsets. We showed that the pre-DC differentiation towards the cDC1 lineage was severely impaired in the BM and the lungs of IAV-infected animals. Ginhoux *et al*. previously reported that Flt3-L is a critical, limiting factor for the cDC1 lineage [22]. In contrast, cDC2 differentiation in the BM (but not in the lungs) of IAV-infected animals was unaffected. It has been reported that M-CSF or GM-CSF act in concert with Flt3-L to promote cDC2 homeostasis in non-lymphoid tissues [22, 69]. In the same line, production of GM-CSF in response to respiratory viral infection (respiratory syncitial virus) rapidly promotes cDC2 differentiation from lung precursors (CD11c^+^MHCII^-^) and cDC2 expansion [70]. It is noteworthy to mention that in this study, this process leads to the further depletion of lung precursors. Along with the decreased Flt3-L production, we also observed a reduced GM-CSF production in the lung during IAV infection; this might also account for the alteration in lung cDC2 lineage. Although further investigations are required, our results suggest that M-CSF and/or GM-CSF are sufficient for the differentiation of pre-DCs towards the cDC2 lineage in the BM of IAV-infected animals.

To the best of our knowledge, a decline in cDC generation associated with a reduced systemic Flt3-L production upon infection has not been reported before. Abrogation of type I and type II IFN signaling did not influence Flt3-L level in the blood and thus argued against a role of these cytokines in the regulation of Flt3-L production. Although the underlying mechanisms are still elusive, an increased apoptosis/cell death of Flt3-L-producing cells (and GM-CSF) during the course of influenza infection may contribute, even partly, to this phenomenon. In line with this hypothesis, expression of Flt3-L in lymphoid organs (spleen, BM, lymph nodes) depends on hematopoietic cells (T, B, NK cells) [71, 72] and IAV was shown to indirectly induce B cell apoptosis in the BM [59]. In the same vein, non-hematopoietic cells are the main producers of Flt3-L in the lungs [71] and apoptosis of epithelial cells consecutive to IAV infection could account for Flt3-L (and GM-CSF) decrease [8, 73]. We showed that inoculation of an Flt3-L-encoding plasmid in mock-treated and IAV-infected mice enhanced the systemic Flt3-L concentration and expanded BM DC progenitors and lung cDCs. Importantly, Flt3-L overexpression leads to similar numbers of DC progenitors in the BM regardless of infection, suggesting that Flt3-L is sufficient to boost the cDC differentiation - mainly the cDC1 pathway - in the BM of infected animals. In contrast, although Flt3-L treatment restored the cDC pool in the lung in IAV-infected mice, the level reached did not attain that of non-infected animals. This suggests that Flt3-L is necessary but that additional factors, *i*.*e*. GM-CSF, are needed to increase the number of pulmonary cDCs.

What are the consequences of the cDC drop during IAV infection? The disrupted differentiation of cDC progenitors described in the present report could account (at least in part) for post-influenza immunosuppression. The drop in the pulmonary cDC number during IAV infection might for instance hamper immune-based therapeutics directed against secondary infections. We have shown that treatment of IAV-infected mice at 7 dpi (but not at 14 dpi) with alpha-galactosylceramide, a potent agonist for invariant natural killer T cells, fails to protect against secondary pneumococcal infection [74]. This failure is at least partly due to a strong decline in the number of cDC1 cells, which are required for invariant natural killer T cell activation [75, 76]. Therefore, strategies designed to prevent cDC depletion in the lungs during IAV infection might be of value in controlling secondary bacterial infections. Previous studies of immunocompromised animals have highlighted the beneficial effects of DC transfer or the enhancement of DC production on susceptibility to secondary systemic or lung infections [47–50]. In these models, the restoration of cDCs enhanced the antibacterial immune response and/or dampened the inflammatory response and the lung injury.

Immunological (defective innate response) and physical (altered pulmonary barrier) mechanisms lead to bacterial outgrowth and dissemination post-influenza [1, 2]. Our data show that Flt3-L overexpression during IAV infection caused a modest decrease of bacterial load in the lungs but significantly lowered lung damage in co-infected mice (reduced alveolitis) and decreased systemic bacterial dissemination. Importantly, these effects were associated with a reduced body weight loss and delayed mortality. Our results argued against a role of Flt3-L on anti-viral response (identical viral burden) and Th17-related anti-pneumococcal response. We rather suggest that Flt-3-L might reinforce the physical barrier in the lungs. In line with this hypothesis, Flt3-L overexpression enhanced the expression of transcripts encoding proteins associated with barrier functions and tissue repair. Mechanistically, the beneficial effect of Flt3-L might be due to the reduced recruitment of CCR2^+^ inflammatory monocytes, a cell population involved in lung epithelial damage and bacterial dissemination post-influenza [7]. How Flt3-L alters the trafficking of this deleterious cell population and whether lung cDCs play a role in this process remain to be investigated. The clinical relevance of our finding is still unclear. Bacterial superinfection post-influenza involves complex mechanisms affecting the antibacterial host defense and the pulmonary barrier function [1, 2, 51]. It is admitted that multiple host (as well as virus and bacteria) targets should be considered for future therapeutic applications [1, 2, 77]. Among them, manipulation of myelopoiesis during influenza infection might represent an interesting option. Emerging evidence indicates that myelopoiesis-triggering factors can be used to prevent severe infections in immunocompromised individuals [78]. As stated above, our present findings indicate a positive effect of Flt3-L overexpression on secondary bacterial infection post-influenza. In the same vein, overproduction of GM-CSF has recently been shown to reverse numeric and functional defects of macrophages during influenza infection and to protect against secondary bacterial infections [12, 79]. Of interest, GM-CSF might also favor barrier functions during IAV infection [79]. We speculate that therapeutic administration of Flt3-L, in association with other growth factors such as GM-CSF, might represent a promising approach for the treatment of secondary bacterial infections post-influenza.

Overall, our results revealed novel mechanisms underlying changes in cDC generation in the context of influenza infection, and suggested that Flt3-L is a potential prognostic marker of susceptibility to secondary infections. Our results also highlighted the potential therapeutic value of Flt3-L in secondary bacterial infections; for example, combining Flt3-L with other growth factors and/or conventional antibacterial drugs (*i*.*e*. antibiotics) might increase therapeutic effectiveness.

## Materials and methods

### Animals and infections

Eight-week-old male wild-type (WT) C57BL/6J mice were purchased from Janvier (Le Genest-St-Isle, France). C57BL/6 *Ifng*^*-/-*^ mice (>10 backcrosses) were from the Jackson laboratory and *Ifnar*^*-/-*^ mice have been described previously [80]. For infection with IAV and *S*. *pneumoniae*, mice were maintained in a biosafety level 2 facility in the Animal Resource Center at the Pasteur Institute, Lille. Mice were anesthetized and administered intranasally (i.n.) with 50μl of PBS containing, or not (mock), 30 plaque forming units (PFUs) of the high-pathogenicity murine-adapted H3N2 IAV strain Scotland/20/74 or 100 PFUs of H1N1 IAV strain WSN/33. For superinfection, mice infected with H1N1 (A/WSN/33) were secondarily infected (4 dpi) with 10^3^ CFUs of *S*. *pneumoniae* serotype 3 (ATCC 6303).

### Ethics statement

Animals were housed in specific pathogen-free environment in Lille Pasteur Institute's animal facilities. Housing and experimentations were carried out according to the French government guidelines of laboratory animal care and approved by the Departmental Direction of Veterinary Services (Prefecture of Lille, France; authorization number: AF 16/20090) and European guidelines of laboratory animal care (European Communities Council Directive of 1986 and revised in 2010, 2010/63/EU). Additionally, the present project has been submitted to, and approved by, the national Institutional Animal Care and Use Committee (CEEA 75) and received the authorization number 00357.03.

### Reagents and antibodies

Monoclonal antibodies (Abs) against mouse CD3 (biotin-conjugated), NK1.1 (biotin-conjugated), Ter119 (biotin-conjugated), CD11b (biotin-conjugated), CD45R/B220 (FITC or biotin-conjugated), Ly6G (FITC-conjugated), Ly6C (APC/Cy7, AlexaFluor 700-conjugated), CD11b (PE/Cy5-conjugated), Ly6A/E (AlexaFluor 647-conjugated) CD19 (FITC-conjugated), CD4 (FITC-conjugated), CD8 (FITC-conjugated), Siglec-F (APC or PE-conjugated), CD45 (Brilliant Violet 510 or APC/Cy7-conjugated), CD117 (Percp-Cy5.5-conjugated), CD135 (APC or PE-conjugated), CD115 (APC or PE-conjugated), CD127 (PE-Cy7-conjugated), CD11c (PE-Cy7 or BV605-conjugated), MHC class II (Pacific-blue or AlexaFluor700-conjugated), CD172α (FITC-conjugated), CD64 (APC-conjugated), Siglec-H (Pacific Blue-conjugated), Siglec-F (PE-conjugated) CD24 (Percp-Cy5.5-conjugated), CD11b (FITC- or biotin conjugated), Ly6C (AlexaFluor700 or APC-Cy7-conjugated), CCR2 (PE-conjugated) and AlexaFluor-700 or PE-conjugated streptavidin were purchased from Ozyme (Montigny le Bretonneux, France) or BD Biosciences (Le Pont de Claix, France). The propidium iodide and annexin V were purchased from Ozyme and BD Biosciences, respectively. Neutralizing monoclonal Abs against IFNγ (R4-6A2), IL-6 (MP5-20F3), TNFα (XT3.11) and isotype control monoclonal Ab (HRPN) were from BioXcell (West Lebanon, NH). Ultrapure LPS (from *Escherichia coli* serotype 0111:B4) was from Invivogen (Toulouse, France). Flt3-L-plasmid was a kind gift from F Andris and O Leo (ULB, Belgium).

### Cell suspensions

Cell suspensions were prepared in PBS/2% FCS. Bone marrow were flushed from both femurs and tibias with cold medium and disrupted with a syringe/needle. The cell suspension was then filtered (90μm) and washed. Spleens were treated with type IV collagenase (1mg/ml) and 1 μg/ml DNase type I (Sigma-Aldrich, Saint Quentin Fallavier, France) at 37°C for 20 min, then disrupted in PBS supplemented with 2% FCS, filtered and washed. Lungs were perfused with PBS, excised and finely minced, followed by enzymatic digestion for 20 min at 37°C in RPMI 1640 containing 1 mg/ml type VIII collagenase (or type IV collagenase for pre-DC analysis) and 1μg/ml DNase type I. After washes, lung homogenates were resuspended in a 20% Percoll and centrifuged at 2000 rpm, without brake, at 22°C for 10 min. After centrifugation, the pellet was washed with medium. For all cell suspensions, red blood cells were removed with lysis buffer (Sigma) and mononuclear cells resuspended in PBS/2%FCS. The total number of cells was determined by trypan blue exclusion.

### Bone marrow-derived DCs

Total BM cells were cultured in 6-well plates at a density of 5 x 10^6^ cells/well in 4ml RPMI/10% FCS, in the presence of Flt3-L (100ng/ml). At day 4, half of the medium was discarded and new medium containing Flt3-L was added. BM-derived DC were used at day 8–9. Phenotypic analysis of BM-derived DC was done by flow cytometry using anti-Siglec H, anti-CD11c, anti-MHCII, anti-CD172α and anti-CD24. For functional analysis, BM-derived DCs were cultured overnight at 1 x 10^5^ cells/well in RPMI/5% SVF in the presence of endotoxin-free LPS (100ng/ml). Cytokine production was measured in the culture supernatants by ELISA.

### Flow cytometry

Cell populations (CD45^+^ cells) were analyzed with a BD LSR Fortessa (BD Biosciences) according to the following cell surface phenotypes: In the BM, (MDPs): Lin(*)^-^CD117^+^CD135^+^CD115^+^CD127^-^; (CDPs) Lin(*)^-^CD117^low^CD135^+^CD115^+^CD127^-^CD11c^-^; (cMoPs): Lin(**)^-^CD117^+^CD135^-^CD115^+^Ly6C^+^CD11b^-^. In the BM, spleen and blood (pre-DCs): Lin(*)^-^CD11c^+^MHCII^-^CD135^+^Sirpα^-/low^ and Siglec-H^-^Ly6C^-^ for cDC1-specific pre-DCs or Siglec-H^-^Ly6C^+^ for cDC2-specific pre-DCs; In the BM and blood, (monocytes): Lin(**)^-^ CD115^+^Ly6G^-^CD11b^+^Ly6C^high^ or Ly6C^low/neg^. In the lungs, (DCs): Siglec-F^-^Ly6G^-^CD11c^+^MHCII^+^ and CD64^-^ for cDCs or CD64^+^ for monocyte-derived DCs; (inflammatory monocytes): Siglec-F^-^Ly6G^-^Ly6C^+^CD11b^+^CCR2^+^CD11c^-^. Dead cells were excluded by propidium iodide staining. For analysis, 10^6^ cells were acquired and the data were analyzed with FACSDiva or FlowJo software (TreeStar, US).

Lin*: CD3, NK1.1, Ter119, CD11b, CD45R/B220, Ly6G

Lin**: CD3, NK1.1, Ter119, CD45R/B220, Ly6G

### Monocyte isolation and restimulation

Bone marrow cells were harvested from naïve or infected mice and cell suspensions were prepared as described above. Monocytes were sorted using a FACSAria III cell sorter (BD Biosciences). Bone marrow cells were labelled with biotin-conjugated anti-Ter119, -CD3, -NK1.1, CD45R/B220 and PE/Cy7-streptavidin, Brilliant Violet 421-anti-CD45 antibody, APC-Cy7-conjugated Ly6G, Alexa Fluor 700-conjugated Ly6C, FITC-conjugated CD11b and PE-conjugated CD115. This protocol yielded >98% cell purity as evaluated by FACS. Then, monocytes were cultured overnight at 1 x 10^5^ cells/well in RPMI/5% SVF in the presence of endotoxin-free LPS (100ng/ml). Cytokine production was measured in the culture supernatants by ELISA.

### Cytokine/growth factor quantification

Levels of IL-6, IL-12p40, and IL-10 were quantified in culture supernatants of TLR agonist-stimulated BM-DCs or monocytes using standardized sandwich enzyme-linked ELISA kits (eBiosciences, R&D systems) and used according to the manufacturer’s recommendations. Similarly, levels of Flt3-L and GM-CSF were quantified by ELISA (R&D systems) in sera as well as in BM and lung tissue lysates. Briefly, total lungs were lysed in 600 μL of lysis buffer (0.2% NP-40, 1 mM EDTA, Anti-protease1X, PBS1X) and incubated for 30 min on ice. The lysates were centrifuged for 30 min at 3500 rpm and the supernatant transferred to a new tube. Bone marrow cells (2x10^7^ cells) were resuspended in 500 μl of radioimmunoprecipitation assay (RIPA) buffer (50 mM tri-HCL, 150 Mm NaCl, 1mM EDTA, 1% Triton, 1% Sodium Deoxycholate, 0,1% SDS, Anti-Protease 1X, PBS 1X) and incubated 15 min on ice with periodic agitation. Lysed cells were centrifuged for 15 min at 13000 rpm at 4°C and the supernatant transferred to a new tube.

### Real-time PCR assays

Total RNAs from whole lungs or BM monocytes were extracted, cDNAs were synthesized and quantitative RT-PCR was carried out. Gene expression was analyzed by the incorporation of the fluorescent dye SYBR green in the double-stranded DNA by using the QuantStudio 12k Flex Software (ThermoFisher Scientific). The murine primers used were as follow: *Cd200* (5’-TTTGCTGTTGTCCCAGGTCCT-3’, 5’-GCATGGCACTGCATTGCTCTA-3’); *Socs3* (5’- GGAACCCTCGTCCGAAGTTC-3’, 5’-CAATCTTCTCGCCCCCACAA-3’); *Il6* (5’- CAACCACGGCCTTCCCTACT-3’, 5’-CCACGATTTCCCAGAGAACATG-3’); *nos2* (5’- GCCACCAACAATGGCAACA-3’, 5’-AGCGTACCGGATGAGCTGTG-3’); *Cx3cr1* (5’- CCTCGTCTTCACGTTCGGTCT-3’, 5’-CACAAAGAGGAGGTCGCTCAA-3’); *Flt3l (*5’- GAGTCAAAAGCCCAGCAGGAT-3’, 5’-GCATTCCTGTCGACGCTAACTT-3’); *Csf2* (5’-TGCCTGTCACGTTGAATGAAGA-3’, 5’-CCCGTAGACCCTGCTCGAATA-3’); *Il17a* (5’-CCGCAATGAAGACCCTGATAGA-3’, 5’-AGAATTCATGTCGTGGTCCAGC-3’); *Il23p19* (5’- CAC-CAG-CGG-GAC-ATA-TGA-AT-3’, 5’- GTT-GTC-CTT-GAG-TCC-TTG-TGG-3’); *Il1b* (5’-TCGTGCTGTCGGACCCATA-3’, 5’-GTCGTTGCTTGGTTCTCCTTGT-3’); *Isg15* (5’-GGCCACAGCAACATCTATGAGG-3’, 5’-CTCGAAGCTCAGCCAGAACTG-3’); *Oas3* (5’-GTGGCACCGATGTCGAACTC-3’, 5’-AGCAACATTCGCATGGCA-3’); *Tjp1* (5’-AGGTCTTCGCAGCTCCAAGAGAAA-3’, 5’-ATCTGGCTCCTCTCTTGCCAACTT-3’); *Ocln* (5’-AGCAGCCCTCAGGTGACTGTTATT-3’, 5’-ACGACGTTAACTCCTGAACAAGCA-3’); *Areg* (5’-TTTGGTGAACGGTGTGGAGAA-3’, 5’-CGAGGATGATGGCAGAGACAA-3’); *Gapdh* (5’-GCAAAGTGGAGATTGTTGCCA -3’, 5’- GCCTTGACTGTGCCGTTGA-3’) were designed using the Primer Express software (Applied Biosystems, Villebon sur Yvette, France). Data were normalized against expression of the *Gapdh* gene and are expressed as a fold-increase over the mean gene expression level in mock-infected mice.

For IAV copy number quantification, reverse transcription was carried out by using primer specific for the viral *M1* gene (5’-TCT AAC CGA GGT CGA AAC GTA-3’), SuperScript II Reverse Transcriptase (ThermoFisher Scientific) and RNAse OUT (ThermoFisher Scientific). A plasmid template harboring the virus M1 negative strand (pPolI-M1, backbone pUC18) was serially diluted (10-folds) to draw a standard curve (Ct value/viral copy number) ranging from 3.8 x10^9^ (Ct ~5) to 380 copies (Ct ~25); allowing virus quantification in samples by interpolation of the Ct values.

### Flt3-L-plasmid injection and infections

C57Bl/6 mice were injected with a Flt3-L- or control (pcDNA3)-plasmid diluted in 1.4 ml of Ringer solution via tail vein as described previously [81]. Plasmid injection was completed in less than 5 sec. Mice were infected with IAV (WSN, 100 PFUs) 24h later. The efficacy of the plasmid-mediated Flt3-L gene delivery in IAV-infected animals was assessed 5 days later. To this end, lung and BM cells were harvested and labelled with a cocktail of antibodies allowing cDC subset and cDC progenitors' identification by flow cytometry. To test the efficacy of the Flt3-L-plasmid delivery on superinfection, IAV-infected mice were superinfected with *S*. *pneumoniae* at 4 dpi. Viable bacteria in the lungs and spleen were counted 36 h after the *S*. *pneumoniae* challenge by plating serial 10-fold dilutions of lung or spleen homogenates onto blood agar plates. The plates were incubated at 37°C overnight and CFUs were counted 24 h later. Survival and body weight were monitored daily after IAV infection and mice were euthanized when they lost in excess of 20% of their initial body weight.

### Assessment of the lung pathology

For histopathologic examination, lungs were fixed by inflation and immersion in PBS 3.2% paraformaldehyde and embedded in paraffin. To evaluate airway inflammation, we subjected fixed lung slices (5 μm sections) to hematoxylin and eosin staining. Three evenly distributed sections per lung were microscopically evaluated by a certified pathologist who was blinded to genotype. To determine the degree of alveolitis, thickness of alveolar wall, alveolar protein exudate, inflammatory cells as well as the distribution of alveolar lesions were scored. The degrees of pulmonary alveolitis were classified into 4 grades. 0: absent; 1: mild; 2: moderate; 3: marked and 4: severe.

### Statistical analysis

All statistical analysis was performed using GraphPad Prism software. Unpaired T-test or one-way ANOVA followed by Bonferroni’s post T-test were used for statistical analysis when two groups or multiple groups were analyzed, respectively. When data did not follow a Gaussian distribution, the statistical significance was evaluated using non-parametric Mann-Whitney *U* or Kruskal-Wallis (followed by a Dunns post-test). We used two-way ANOVA when multiple parameters were studied. For body weight evolution, means of “area under the curve” of each individual were compared using a Mann–Whitney U test. The survival of infected mice was analyzed using the Kaplan–Meier method and a log-rank test. *, p < 0.05; **, p < 0.01; ***, p < 0.001.

## Supporting information

S1 Fig (A) Mice were infected i.n. with H3N2 virus. Gating strategy to analyze total lung cDCs (Siglec-F-CD11c+MHCII+CD64-) and cDC subsets (CD172α+CD24+ and CD172α-CD24++) at day 0 (left panel) and 4dpi (right panel) is shown. (B) Gating strategy to analyze BM MDPs (Lin-CD117+CD135+CD115+), CDPs (Lin-CD117lowCD135+CD11c-CD115+) and pre-DCs (Lin-CD11c+MHCII-CD135+) is shown.
(TIF)

S2 Fig Gating strategy to analyze spleen and lung pre-DCs. Lineage cocktail: Ter119, CD3, NK1.1, CD45RB220, Ly6G is shown.
(TIF)

S3 Fig (A) Gating strategy to analyze Flt3-L-generated BM DC subsets is shown. The remaining cells (CD11c-MHCII-) were CD11b-CD115-. (B) BM-derived cells in the presence of Flt3-L, were stimulated with LPS (100ng/ml) for 24h and IL-10 was quantified in the supernatant. Means ± SEM of 4 mice are represented.
(TIF)

S4 Fig Mice were i.v. injected with 2μg of a control- or Flt3-L- encoding plasmid and 24h later were infected or not with IAV. Four days later, the mean of fluorescent intensity (MFI) of CD135 expression on BM pre-DCs (*left panel*) and the number of lung neutrophils (*right panel*) were analyzed by flow cytometry. Means ± SEM of 6–12 mice are represented. **, p < 0.01; ***, p < 0.001.
(TIF)
